# Patterns and early evolution of organ failure in the intensive care unit and their relation to outcome

**DOI:** 10.1186/cc11868

**Published:** 2012-11-16

**Authors:** Yasser Sakr, Suzana M Lobo, Rui P Moreno, Herwig Gerlach, V Marco Ranieri, Argyris Michalopoulos, Jean-Louis Vincent, the SOAP Investigators

**Affiliations:** 1 Department of Anesthesiology and Intensive Care, Friedrich-Schiller-University, Erlanger Allee 103, 07743 Jena, Germany; 2 Division of Critical Care Medicine, Department of Internal Medicine, Medical School-FUNFARME and Hospital de Base, São José do Rio Preto, São Paulo, Brazil; 3 Dunidade de Cuidados Intensivos Neurocriticos, Hospital de São José, Centro Hospitalar de Lisboa Central, E.P.E., Rua José António Serrano, 1150-199 Lisbon, Portugal; 4 Department of Anesthesia, Intensive Care Medicine, and Pain Management, Vivantes-Klinikum Neukölln, Rudower Strasse 48, D-12313 Berlin, Germany; 5 Department of Anesthesia and Critical Care, Ospedale S. Giovanni Battista-Molinette, corso Dogliotti 14, 10126, University of Turin, Turin, Italy; 6 Intensive Care Unit, Henry Dunant Hospital, Department of Medicine, Athens, Greece; 7 Department of Intensive Care, Erasme Hospital, Université Libre de Bruxelles, Brussels, Route de Lennik 808, 1070 Brussels, Belgium

## Abstract

**Introduction:**

Recognition of patterns of organ failure may be useful in characterizing the clinical course of critically ill patients. We investigated the patterns of early changes in organ dysfunction/failure in intensive care unit (ICU) patients and their relation to outcome.

**Methods:**

Using the database from a large prospective European study, we studied 2,933 patients who had stayed more than 48 hours in the ICU and described patterns of organ failure and their relation to outcome. Patients were divided into three groups: patients without sepsis, patients in whom sepsis was diagnosed within the first 48 hours after ICU admission, and patients in whom sepsis developed more than 48 hours after admission. Organ dysfunction was assessed by using the sequential organ failure assessment (SOFA) score.

**Results:**

A total of 2,110 patients (72% of the study population) had organ failure at some point during their ICU stay. Patients who exhibited an improvement in organ function in the first 24 hours after admission to the ICU had lower ICU and hospital mortality rates compared with those who had unchanged or increased SOFA scores (12.4 and 18.4% versus 19.6 and 24.5%, *P *< 0.05, pairwise). As expected, organ failure was more common in sepsis than in nonsepsis patients. In patients with single-organ failure, in-hospital mortality was greater in sepsis than in nonsepsis patients. However, in patients with multiorgan failure, mortality rates were similar regardless of the presence of sepsis. Irrespective of the presence of sepsis, delta SOFA scores over the first 4 days in the ICU were higher in nonsurvivors than in survivors and decreased significantly over time in survivors.

**Conclusions:**

Early changes in organ function are strongly related to outcome. In patients with single-organ failure, in-hospital mortality was higher in sepsis than in nonsepsis patients. However, in multiorgan failure, mortality rates were not influenced by the presence of sepsis.

## Introduction

Multiple organ failure (MOF) is an evolving clinical syndrome triggered by various stimuli and may be a consequence of tissue hypoperfusion with cellular hypoxia, metabolic dysfunction, and impaired bioenergetic processes [1]. MOF is the main cause of morbidity and mortality in patients admitted to the intensive care unit (ICU) and is recognized as the final common pathway preceding death in critically ill patients [2-4]. Sepsis, a major public health problem, often progresses to MOF [2,3], which is believed to increase markedly the risk of death in ICU patients [2,3,5].

A need exists to evaluate organ function better over time in ICU patients. The Sequential Organ Failure Assessment (SOFA) score [6-8] was developed as a tool to describe quantitatively the time course of organ dysfunction [9,10]. Changes in SOFA score have been correlated with prognosis (delta SOFA and SOFA max) [8,10] and are now widely used to assess the effects of therapeutic interventions [11,12]. The aim of our study was to investigate the relation between the patterns of early changes in organ function in the ICU and outcome from critical illness. The study set up the hypothesis that recognition of early changes in organ function may characterize the clinical course of critically ill patients. Moreover, we explored the relative roles of sepsis and MOF in determining outcome in critically ill patients.

## Materials and methods

This study is a subanalysis of the prospective, multicenter, observational study, the sepsis occurrence in acutely ill patients (SOAP) study, which was designed to create a database of ICU patients in European countries. Recruitment, data collection, and management are detailed elsewhere [2]; in brief, all patients older than 15 years admitted to the 198 participating centers (see the Acknowledgements for a list of participating countries and centers) between May 1 and May 15, 2002, were included. Patients who stayed in the ICU for less than 24 hours for routine postoperative observation were not included. Patients were followed up until death, hospital discharge, or for 60 days. Because the observational SOAP study did not require any deviation from routine medical practice, institutional review board approval was either waived or expedited in participating institutions, and informed consent was not required. As such, no supplementary review-board documents were needed for the current substudy.

Data were collected prospectively by using preprinted case-report forms. Data collection on admission included demographic data and comorbidities. Clinical and laboratory data for the simplified acute physiology score (SAPS) II [13] were reported as the worst value within 24 hours after admission. Microbiologic and clinical infections were reported daily, as well as the antibiotics administered. Organ function was evaluated on admission and daily thereafter, by using the SOFA score [6]. Data were encoded centrally in the organizing center by medical personnel (Department of Intensive Care, Erasme Hospital), and a number of quality control measures were carried out to assure consistency of the data and the quality of data entry [2]. All variables were defined *a priori*, and definitions were available on an Internet-based website throughout the study period.

### Definitions

Sepsis syndromes were defined according to consensus conference definitions [14]. Organ failure was defined as a SOFA score >2 for any of the six organs/systems evaluated, and MOF, as more than one failing organ [7]. An increase in the SOFA score of at least 1 point was considered as deterioration in organ function. SOFAmax was defined as the maximum SOFA score recorded during the ICU stay, and SOFAmean, as the mean value during the ICU stay. Individual organ failures during the ICU stay were defined according to the SOFAmax for the corresponding organ. Combinations of organ failures were considered independent of the time of onset of each organ failure. The delta SOFA (ΔSOFA) was calculated as the difference between the SOFA score on a specific day and the score on the day of admission to the ICU [8].

### Statistical analysis

For the purposes of this analysis, we excluded all patients who spent less than 48 hours in the ICU. To investigate the impact of sepsis on organ failure, patients were divided into three groups; Patients without sepsis, those in whom sepsis developed within 48 hours after ICU admission, and those in whom sepsis developed more than 48 hours after admission to the ICU.

Data were analyzed by using SPSS 13.0 for Windows (SPSS Inc., Chicago, IL, USA). Descriptive statistics were computed for all study variables. The Kolmogorov-Smirnov test was used to verify the normality of distribution of continuous variables. Nonparametric tests of comparison were used for variables evaluated as not normally distributed. Difference testing between groups was performed by using the two-tailed *t *test, Mann-Whitney *U *test, χ^2 ^test, and Fisher Exact test, as appropriate, with a Bonferroni correction for multiple comparisons. Differences in SOFA scores between groups over time were assessed by using multifactorial analysis of variance (ANOVA).

Continuous data are presented as mean ± SD, and categoric data, as number (%), unless otherwise indicated. All statistics were two-tailed, and a *P *< 0.05 was considered to be statistically significant.

## Results

## Characteristics of the study group

Of the 3,147 patients included in the SOAP database, 2,933 patients stayed in the ICU for more than 48 hours (Table 1). The admission SOFA score for these patients was 5.0 ± 3.7; the maximum SOFA, 6.6 ± 4.4; and the mean SOFA, 5.0 ± 3.7. The median ICU length of stay was 3.4 (IQ, 2.0 to 7.6) days, and the median length of hospital stay, 16 (8 to 33) days. The overall ICU and hospital mortality rates were 16.6% and 21.9%, respectively.

**Table 1:** Characteristics of the patients on ICU admission, lengths of ICU and hospital stays, and outcomes

Number of patients	2,933
Age, years, median (IQ)^a^	64 (50-74)

Sex, male/female, %	61.5/38.5

Type of admission, n (%)	
Medical	1,584 (54.0)
Surgical	1,349 (46.0)
Elective	766 (26.1)
Emergency	583 (19.9)

ICU admission source, n (%)^b^	
ER/ambulance	818 (27.9)
Hospital floor	726 (24.8)
OR/recovery room	765 (26.1)
Other hospital	332 (11.3)

Comorbidities, n (%)	
COPD	322 (11.0)
Cancer	299 (10.2)
Heart failure	287 (9.8)
Diabetes	208 (7.1)
Cirrhosis	115 (3.9)
Hematologic cancer	63 (2.1)
HIV/AIDS	8 (0.3)

SAPS II score, mean ± SD	35.9 ± 16.4

SOFA score, mean ± SD	5.0 ± 3.7

Duration of ICU stay, days, median (IQ)	3.4 (2.0-7.6)

Duration of hospital stay, days, median (IQ)	16.0 (8.0-33.0)

ICU mortality, n (%)^c^	486 (16.6)

Hospital mortality, n (%)^d^	642 (21.9)

^a^Seven missing values; ^b^292 missing values; ^c^one missing value; ^d^44 missing values.

### Patterns of organ failure and their relation to outcome

On admission to the ICU, 1,675 (57.1%) patients had at least one organ failure; these patients had ICU and hospital mortality rates of 27.6% and 34.2%, respectively. In total, 435 (15%) patients developed organ failure in the ICU. The organ failures most commonly present on the day of admission to the ICU were of the cardiovascular (24%) and respiratory (22%) systems, whereas respiratory (43%) and renal (36%) organ failures were the most prevalent during the ICU stay (Table 2). The combination of respiratory and cardiovascular organ failures was the most common on admission (9%) and during the ICU stay (25%).

**Table 2:** ICU and hospital mortality rates according to the number, type, and combinations of failed organs

	On admission to the ICU			At any time during ICU stay		
	
	Incidence (%)	Mortality (%)		Incidence (%)	Mortality (%)	
				
		ICU	Hospital		ICU	Hospital
Number of failed organs						

1	927 (32)	17	23	942 (32)	5	11
2	524 (18)	28	37	677 (23)	24	33
3	181 (6)	45	50	334 (11)	44	51
>3	43 (1)	58	70	157 (5)	73	79

Type of organ failure^a^						

Cardiovascular	717 (24)	31	39	993 (34)	35	41
Respiratory	653 (22)	27	33	1,258 (43)	29	36
CNS	601 (21)	32	39	757 (26)	38	45
Renal	513 (18)	26	35	1,058 (36)	28	36
Coagulation	130 (18)	36	45	290 (10)	44	54
Hepatic	82 (3)	33	40	165 (6)	38	45

Combinations of two organ failures						

Respiratory + cardiovascular	274 (9)	39	46	726 (25)	39	46
Respiratory + renal	120 (4)	42	49	543 (19)	41	48
Respiratory + CNS	197 (7)	38	47	488 (17)	43	50
Hepatic + renal	24 (1)	38	46	98 (3)	47	51
Respiratory + hepatic	22 (1)	50	50	109 (4)	47	53
Renal + cardiovascular	24 (1)	38	46	481 (16)	47	55
CNS + cardiovascular	220 (8)	39	48	390 (13)	50	55
CNS + renal	109 (4)	39	51	308 (11)	49	57
Hepatic + cardiovascular	29 (1)	48	52	97 (3)	55	59
Respiratory + coagulation	34 (1)	47	53	203 (7)	53	63
Coagulation + cardiovascular	51 (2)	47	59	201 (7)	56	65
Coagulation + renal	25 (1)	52	68	159 (5)	59	67
Coagulation + hepatic	15 (1)	67	80	64 (2)	63	70
Hepatic + CNS	29 (1)	58	67	97 (3)	66	71
Coagulation + CNS	23 (1)	65	74	113 (4)	69	79

^a^Alone or in combination with other organs.

**Figure 1:**
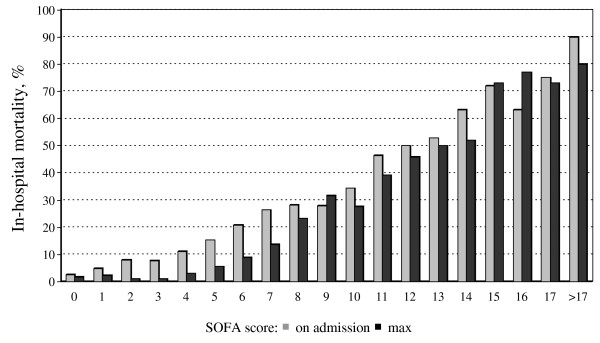
**Hospital mortality rates according to SOFA score on admission (gray columns) and the maximum SOFA score (black columns)**.

Hospital mortality rates increased according to the severity of organ dysfunction/failure as assessed by the SOFA score (Figure 1) and according to the number of failing organs (Table 2). The highest hospital mortality rates were observed in patients with failure of the hepatic or coagulation systems, and in patients with combined coagulation and hepatic or coagulation and central nervous system (CNS) failure, mortality rates reached 70% to 80%.

### Early changes in organ failure and outcome

**Figure 2:**
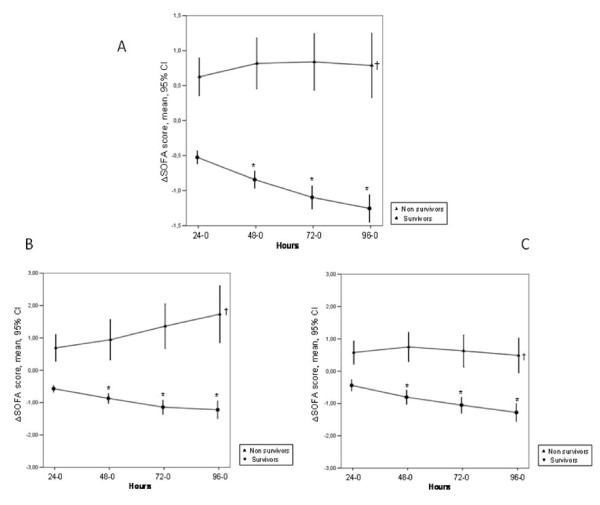
**Error bars representing the delta SOFA scores (mean ± 95% CI) during the first 4 days in the ICU in survivors (solid circles) and nonsurvivors (solid triangles) in the whole cohort (A) (*n *= 2,933) and in patients without (*n *= 1,789) (B), or with sepsis (*n *= 1,144) (C)**. *P < 0.05 compared with ΔSOFA 24-0 (ANOVA with Bonferroni correction for multiple comparisons); †multifactorial ANOVA; *P *< 0.05 compared with survivors.

The time to achieve SOFAmax was longer in nonsurvivors than in survivors (2 (2 to 3) versus 1 (1 to 2) days; *P *< 0.001). Patients who exhibited an improvement or no change in organ function over the first 24 hours after admission to the ICU had lower ICU and hospital mortality rates compared with those whose scores increased (12.4 and 18.4% versus 19.6 and 24.5%; *P *< 0.05 pairwise). Likewise, patients who exhibited an improvement or no change in organ function over the second day in the ICU had lower ICU and hospital mortality rates compared with those whose scores increased (13.8 and 20.7% versus 18.6 and 24.1%; *P *< 0.05 pairwise). Delta SOFA scores were higher and remained higher in nonsurvivors than in survivors over the first 4 days in the ICU; they decreased significantly over time in survivors (Figure 2).

## Impact of sepsis on incidence and outcome from organ failure

In total, 1,144 (39%) patients had sepsis at some point during the ICU stay, including 865 during the first 48 hours in the ICU and 279 after 48 hours. MOF occurred more frequently in patients with sepsis, irrespective of the time of onset (Table 3). During the ICU stay, renal failure was the most common organ failure in patients who never had sepsis, and respiratory failure was the most common in patients with sepsis.

**Table 3:** Incidence of organ failure according to the presence of sepsis

	**No sepsis (***n ***= 1,789)**	Sepsis within 48 hours (*n *= 865)	Sepsis after 48 hours (*n *= 279)
Organ failure on admission to the ICU			

Number of failing organs, median (IQ)	0 (0-1)	1 (0-2)^a^	1 (0-2)^a^
Any organ failure, *n *(%)	874 (49)	607 (70)^a^	194 (70)^a^
1	563 (32)	271 (31)	93 (33)
2	228 (13)	219 (25)^a^	77 (28)^a^
3	72 (4)	87 (10)^a^	22 (8)^a^
>3	11 (1)	30 (3)^b^	2 (1)
Type of organ failure			
Respiratory	320 (18)	256 (30)^a^	77 (28)^a^
CNS	317 (18)	199 (23)^a^	85 (31)^a^
Cardiovascular	306 (17)	320 (37)^a^	91 (33)^a^
Renal	258 (14)	210 (24)^a^	45 (16)
Coagulation	49 (3)	69 (8)^a^	12 (4)
Hepatic	30 (2)	41 (5)^a^	11 (4)^b^

Organ failure at any time during ICU stay			

Number of failing organs, median (IQ)	1 (0-2)	2 (1-3)	2 (1-3)
Any organ failure, *n *(%)	1,120 (63)	732 (85)^a^	258 (92)^a^
1	652 (36)	230 (27)^a^	60 (22)^a^
2	307 (17)	253 (29)^a^	117 (42)^a^
3	121 (7)	158 (18)^a^	55 (20)^a^
>3	40 (2)	98 (11)^a^	26 (9)^a^
Type of organ failure			
Renal	559 (31)	386 (45)^a^	113 (41)^a^
Respiratory	514 (29)	528 (61)^a^	216 (77)^a^
Cardiovascular	406 (23)	432 (50)^a^	155 (56)^a^
CNS	368 (21)	270 (31)^a^	119 (43)^a^
Coagulation	102 (6)	143 (17)^a^	45 (16)^a^
Hepatic	49 (3)	84 (10)^a^	32 (12)^a^

CNS, central nervous system; IQ, interquartile range. ^b^*P *< 0.05; ^a^*P *< 0.01 compared with patients without sepsis (pairwise comparisons with Bonferroni correction for multiple comparisons).

ICU (24 and 28 versus 11%; *P *< 0.001 each) and hospital mortality rates (33 and 33 versus 14%; *P *< 0.001 each) were more than double in patients who had sepsis within or after 48 hours than in those who never had sepsis. Mortality from any type of organ failure was higher in patients with sepsis than in those who did not have sepsis, irrespective of the time of onset (Table 4). Hospital mortality rates in patients with single-organ failure during the ICU stay were higher in patients with sepsis than in those who never had sepsis (sepsis within 48 hours and sepsis after 48 hours versus no sepsis: hospital mortality, 16 and 13 versus 9%, respectively; *P *< 0.01 pairwise). However, patients with MOF had similar mortality rates regardless of whether they had sepsis (ICU mortality ranging from 23% to 76%, and hospital mortality ranging from 32% to 89%).

**Table 4:** ICU and hospital mortality rates according to the number and type of failed organs and the presence of sepsis

	ICU mortality (%)			Hospital mortality		
	
	No sepsis *n *= 1,789	Sepsis within 48 hours *n *= 865	Sepsis after 48 hours *n *= 279	No sepsis *n *= 1,789	Sepsis within 48 hours *n *= 865	Sepsis after 48 hours *n *= 279
On admission to the ICU						
Any organ failure	20	30^a^	30^a^	25	40^a^	37^a^
1	13	20^a^	30^a^	18	30^a^	36^a^
2	25	30	29	33	41^a^	38^a^
3	44	48	32	47	56	36
> 3	55	60	50	64	73	50
Type of organ failure						
Respiratory	10	34^a^	31^a^	25	44^a^	36^a^
CNS	34	32	27^a^	38	42^b^	36^a^
Cardiovascular	25	38^a^	28^b^	31	48^a^	33^a^
Renal	20	32^a^	31^a^	26	44^a^	42^a^
Coagulation	22	46^a^	33^b^	27	58^a^	50^b^
Hepatic	20	34^a^	64^a^	28	42^a^	64^a^

At any time during the ICU stay						

Any organ failure	17	28^a^	30^a^	22	38^a^	37^a^
1	5	6	7	9	16^a^	13^a^
2	24	23	27	32	34	34
3	47	43	42	50	52	51
>3	76	71	69	76	89	77
Type of organ failure						
Renal	20	38^a^	41^a^	26	50^a^	50^a^
Respiratory	24	32^a^	32^a^	28	41^a^	40^a^
Cardiovascular	29	40^a^	37^a^	33	49^a^	42^a^
CNS	36	40^a^	39^a^	40	50^a^	46^a^
Coagulation	33	49^a^	53^a^	41	60^a^	64^a^
Hepatic	27	42^a^	47^a^	33	49^a^	50^a^

CNS, central nervous system; IQ, interquartile range. ^b^*P *< 0.05; ^a^*P *< 0.01 compared with patients without sepsis (pairwise comparisons with Bonferroni correction for multiple comparisons).

Delta SOFA scores over the first 4 days in the ICU were higher in nonsurvivors than in survivors, regardless of the presence of sepsis (Figure 2). SOFA scores remained elevated in survivors but decreased over time in survivors, irrespective of the presence of sepsis.

## Discussion

The main findings of our study were that (a) whatever the degree of organ failure at ICU admission, patients who exhibited an improvement or no change in organ function during the first 24 to 48 hours in the ICU had lower mortality rates than did those in whom organ function worsened; (b) in patients with single-organ failure during the ICU stay, hospital mortality was significantly greater in patients with sepsis than in those without; (c) patients with MOF had similar mortality rates, regardless of the presence of sepsis.

The association between early improvement in organ function and favorable prognosis has been reported in several other studies [8,15,16]. In 287 patients with severe sepsis, Russell *et al. *[15] reported that worsening of organ function over the first 3 days after the onset of sepsis syndrome was associated with higher 30-day mortality rates than was improvement or no change in organ function. In a study of 1,036 patients with severe sepsis, Levy *et al. *[16] also reported that early changes (baseline to day 1) in organ function were closely related to outcome. Interestingly, we found that the evolution of organ function on the second day in the ICU was associated with outcome, irrespective of the degree of organ function on admission to the ICU. These observations may help to identify patients in whom continuing therapy is likely to be futile [17], or to define patients who may benefit from a change in therapeutic strategy (for example, another surgical intervention, a change in antibiotic therapy, or more-intensive vasoactive support).

We found that the time required to achieve the highest degree of organ dysfunction/failure was shorter in survivors than in non-survivors. However, no specific pattern of organ failure was related to the presence of sepsis in our patients. Dulhunty *et al. *[18] reported that CNS dysfunction was more commonly present in patients with systemic inflammatory response syndrome (SIRS) but no infection and was associated more commonly with death in these patients than in those with sepsis. However, the assessment of neurologic failure using the GCS may be confounded by the frequent use of sedative agents in critically ill patients.

Several studies [3,6,16,19] have investigated the epidemiology and outcome of sepsis-associated organ dysfunction/failure; however, studies in the nonsepsis population are scarce [4,18]. Dulhunty *et al. *[18] investigated the time course of organ dysfunction and outcome in patients with severe sepsis and patients with severe noninfectious SIRS, but excluded all other patients, which may explain the higher ICU mortality rate of 25% in their noninfected population, compared with the 20% in our study. In our study, hospital mortality from single-organ failure during the ICU stay was greater in patients with sepsis than in those who never had sepsis; interestingly, mortality rates from MOF were similar, regardless of the presence of sepsis. In their large cohort of ICU patients in Australasia, Dulhunty *et al. *[18] reported similar findings.

Interestingly, the highest hospital mortality rates were observed in patients with failure of the hepatic or coagulation systems. Because disturbances of coagulation parameters are closely related with liver failure, this study highlights the enormous importance of liver function on the outcome of ICU patients. Umegaki *et al. *[20] also recently reported that hepatic dysfunction compared with other organ dysfunction was associated with the highest mortality rates in a study including 4,196 patients with severe sepsis.

A key strength of our study is the large database of patients and its multicenter, pan-European nature. The SOAP study was performed several years ago, but organ-dysfunction patterns are likely to change slowly over time, so these data are still relevant. Nevertheless, our study has some limitations. First, participation was on a voluntary basis. Second, our results can be extrapolated only to ICUs with a similar case-mix. Third, other factors, which were not considered in our report, may influence the outcome of MOF, including comorbidities, severity of illness, and local practice. We also did not discriminate between acute and chronic organ failure in our analysis.

## Conclusions

Although sepsis patients have worse outcomes than do nonsepsis patients, the differences are primarily in patients with only one organ failure, as the mortality in MOF is very high, regardless of the presence of sepsis. In all patients (with or without sepsis), changes in organ function during the first 24 to 48 hours after ICU admission can determine outcome, irrespective of the baseline degree of organ dysfunction. These patterns of organ failure and their relation to outcome may be useful in prognostication and, hence, in risk stratification of critically ill patients, including in the setting of clinical trials.

## Key messages

• Whatever the degree of organ failure on admission to the ICU, patients who exhibited an improvement in organ function during the first 24 to 48 hours in the ICU had lower mortality rates than did the other patients.

• Higher mortality rates in sepsis compared with nonsepsis patients are primarily the result of higher mortality associated with single rather than multiorgan failure.

• Mortality rates in patients with multiorgan failure are high, irrespective of the presence of sepsis.

• Improved knowledge of patterns of organ failure and their relation to outcome may be useful in prognostication and, hence, in risk stratification of critically ill patients.

## Abbreviations

CNS: central nervous system; GCS: Glasgow Coma Scale; ICU: intensive care unit; MOF: multiple organ failure; SAPS: simplified acute physiology score; SIRS: systemic inflammatory response syndrome; SOFA: sequential organ failure assessment.

## Competing interests

The authors declare that they have no competing interests.

## Authors' contributions

JLV conceived the initial SOAP study. RM, HG, VMR, YS, and JLV participated in the design and coordination of the SOAP study. YS performed the statistical analyses. SL, YS, and JLV drafted the present manuscript. RM, VMR, AG, YS revised the draft. All authors read and approved the final manuscript.

## References

[B1] Seely AJ Christou NV Multiple organ dysfunction syndrome: exploring the paradigm of complex nonlinear systems Crit Care Med 2000 16 2193 2200 10921540 10.1097/00003246-200007000-00003

[B2] Vincent JL Sakr Y Sprung CL Ranieri VM Reinhart K Gerlach H Moreno R Carlet J Le Gall JR Payen D Sepsis in European intensive care units: results of the SOAP study Crit Care Med 2006 16 344 353 10.1097/01.CCM.0000194725.48928.3A 16424713

[B3] Angus DC Linde-Zwirble WT Lidicker J Clermont G Carcillo J Pinsky MR Epidemiology of severe sepsis in the United States: analysis of incidence, outcome, and associated costs of care Crit Care Med 2001 16 1303 1310 10.1097/00003246-200107000-00002 11445675

[B4] Afessa B Green B Delke I Koch K Systemic inflammatory response syndrome, organ failure, and outcome in critically ill obstetric patients treated in an ICU Chest 2001 16 1271 1277 10.1378/chest.120.4.1271 11591571

[B5] Martin GS Mannino DM Eaton S Moss M The epidemiology of sepsis in the United States from 1979 through 2000 N Engl J Med 2003 16 1546 1554 10.1056/NEJMoa022139 12700374

[B6] Vincent JL Moreno R Takala J Willatts S De Mendonca A Bruining H Reinhart CK Suter PM Thijs LG The SOFA (Sepsis-related Organ Failure Assessment) score to describe organ dysfunction/failure, On behalf of the Working Group on Sepsis-Related Problems of the European Society of Intensive Care Medicine Intensive Care Med 1996 16 707 710 10.1007/BF01709751 8844239

[B7] Vincent JL De Mendonca A Cantraine F Moreno R Takala J Suter PM Sprung CL Colardyn F Blecher S Use of the SOFA score to assess the incidence of organ dysfunction/failure in intensive care units: results of a multicenter, prospective study; Working group on "sepsis-related problems" of the European Society of Intensive Care Medicine Crit Care Med 1998 16 1793 1800 10.1097/00003246-199811000-00016 9824069

[B8] Ferreira FL Bota DP Bross A Melot C Vincent JL Serial evaluation of the SOFA score to predict outcome in critically ill patients JAMA 2001 16 1754 1758 10.1001/jama.286.14.1754 11594901

[B9] Antonelli M Moreno R Vincent JL Sprung CL Mendoca A Passariello M Riccioni L Osborn J Application of SOFA score to trauma patients: Sequential Organ Failure Assessment Intensive Care Med 1999 16 389 394 10.1007/s001340050863 10342513

[B10] Moreno R Vincent JL Matos R Mendonca A Cantraine F Thijs L Takala J Sprung C Antonelli M Bruining H Willatts S The use of maximum SOFA score to quantify organ dysfunction/failure in intensive care: results of a prospective, multicentre study; Working Group on Sepsis related Problems of the ESICM Intensive Care Med 1999 16 686 696 10.1007/s001340050931 10470572

[B11] Cruz DN Antonelli M Fumagalli R Foltran F Brienza N Donati A Malcangi V Petrini F Volta G Bobbio Pallavicini FM Rottoli F Giunta F Ronco C Early use of polymyxin B hemoperfusion in abdominal septic shock: the EUPHAS randomized controlled trial JAMA 2009 16 2445 2452 10.1001/jama.2009.856 19531784

[B12] Jabre P Combes X Lapostolle F Dhaouadi M Ricard-Hibon A Vivien B Bertrand L Beltramini A Gamand P Albizzati S Perdrizet D Lebail G Chollet-Xemard C Maxime V Brun-Buisson C Lefrant JY Bollaert PE Megarbane B Ricard JD Anguel N Vicaut E Adnet F Etomidate versus ketamine for rapid sequence intubation in acutely ill patients: a multicentre randomised controlled trial Lancet 2009 16 293 300 10.1016/S0140-6736(09)60949-1 19573904

[B13] Le Gall JR Lemeshow S Saulnier F A new Simplified Acute Physiology Score (SAPS II) based on a European/North American multicenter study JAMA 1993 16 2957 2963 10.1001/jama.1993.03510240069035 8254858

[B14] Bone RC Balk RA Cerra FB Dellinger RP Fein AM Knaus WA Schein RM Sibbald WJ Definitions for sepsis and organ failure and guidelines for the use of innovative therapies in sepsis: the ACCP/SCCM Consensus Conference Committee, American College of Chest Physicians/Society of Critical Care Medicine Chest 1992 16 1644 1655 10.1378/chest.101.6.1644 1303622

[B15] Russell JA Singer J Bernard GR Wheeler A Fulkerson W Hudson L Schein R Summer W Wright P Walley KR Changing pattern of organ dysfunction in early human sepsis is related to mortality Crit Care Med 2000 16 3405 3411 10.1097/00003246-200010000-00005 11057793

[B16] Levy MM Macias WL Vincent JL Russell JA Silva E Trzaskoma B Williams MD Early changes in organ function predict eventual survival in severe sepsis Crit Care Med 2005 16 2194 2201 10.1097/01.CCM.0000182798.39709.84 16215369

[B17] Cabre L Mancebo J Solsona JF Saura P Gich I Blanch L Carrasco G Martin MC Multicenter study of the multiple organ dysfunction syndrome in intensive care units: the usefulness of Sequential Organ Failure Assessment scores in decision making Intensive Care Med 2005 16 927 933 10.1007/s00134-005-2640-2 15856171

[B18] Dulhunty JM Lipman J Finfer S Does severe non-infectious SIRS differ from severe sepsis? Results from a multi-centre Australian and New Zealand intensive care unit study Intensive Care Med 2008 16 1654 1661 10.1007/s00134-008-1160-2 18504549

[B19] Guidet B Aegerter P Gauzit R Meshaka P Dreyfuss D Incidence and impact of organ dysfunctions associated with sepsis Chest 2005 16 942 951 10.1378/chest.127.3.942 15764780

[B20] Umegaki T Ikai H Imanaka Y The impact of acute organ dysfunction on patients' mortality with severe sepsis J Anaesthesiol Clin Pharmacol 2011 16 180 184 10.4103/0970-9185.81816 21772676 PMC3127295

